# Assessment of the Extrusion Process and Printability of Suspension-Type Drug-Loaded Affinisol^TM^ Filaments for 3D Printing

**DOI:** 10.3390/pharmaceutics14040871

**Published:** 2022-04-15

**Authors:** Gloria Mora-Castaño, Mónica Millán-Jiménez, Vicente Linares, Isidoro Caraballo

**Affiliations:** Department of Pharmacy and Pharmaceutical Technology, Faculty of Pharmacy, Universidad de Sevilla, 41012 Seville, Spain; gmora1@us.es (G.M.-C.); momillan@us.es (M.M.-J.); vlinares@us.es (V.L.)

**Keywords:** extrusion process, 3D printing, fused-deposition modelling, additive manufacturing, drug-loaded filaments, printability, critical points, fractal dimension

## Abstract

Three-dimensional (3D) printing technology enables the design of new drug delivery systems for personalised medicine. Polymers that can be molten are needed to obtain extruded filaments for Fused Deposition Modelling (FDM), one of the most frequently employed techniques for 3D printing. The aim of this work was to evaluate the extrusion process and the physical appearance of filaments made of a hydrophilic polymer and a non-molten model drug. Metformin was used as model drug and Affinisol™ 15LV as the main carrier. Drug-loaded filaments were obtained by using a single-screw extruder and, subsequently, their printability was tested. Blends containing up to a 60% and 50% drug load with 5% and 7.5% of auxiliary excipients, respectively, were successfully extruded. Between the obtained filaments, those containing up to 50% of the drug were suitable for use in FDM 3D printing. The studied parameters, including residence time, flow speed, brittleness, and fractal dimension, reflect a critical point in the extrusion process at between 30–40% drug load. This finding could be essential for understanding the behaviour of filaments containing a non-molten component.

## 1. Introduction

Three-dimensional printing technology has undergone rapid growth over recent years. In the pharmaceutics industry field, the research has been pumped by the approval of the FDA (Food and Drug Administration) in 2015 of the first drug produced by 3D printing, Spritam [1,2].

Three-dimensional printing brings advantages in drug production because it allows the building of objects with high precision and accuracy [3]. Several delayed-release, sustained-release, and rapid/immediate-release dosage forms have been produced, such as implants, polypills, tablets, capsules, etc. [2].

There are several types of 3D printing technologies applicable for pharmaceutical development, Fused Deposition Modelling (FDM) being one of the most frequently employed. In FDM printers, thermoplastic polymeric filaments advance to a heating zone where they are melted, and afterwards, they are extruded through a heating nozzle and deposed on a printing platform, layer by layer, undergoing instant solidification until the object is formed. This allows for the production of dosage forms with high-complexity geometries, being able to customize the formulation by varying parameters such as the dimensions, the fill density, and the percentage of drug in the dosage. FDM also allows for different drug dissolution rates and profiles to be obtained, making it an ideal technique for individualised therapies. Filaments containing a drug can be also used in FDM. The drug’s incorporation into the filament can be carried out through two different methods: filament impregnation with the drug or Hot Melt Extrusion (HME) [2,3,4,5].

Hot Melt Extrusion (HME) technology is a continual process that applies heat and pressure to melt material and can be used to generate filaments in a uniform way, being able to change the physical properties of the materials. However, the processing conditions have to be adequately optimized. Extrusion machines are composed of a motor, an extrusion cylinder, one or two turning screws, a heater, and a die. There are two main types of hot-melt extruders: the single-screw extruder and the twin-screw extruder. In the single-screw extruder, high pressure may be created during the melting process, which could cause poor mixing. Thus, mixing prior to extrusion should be performed when using single-screw extruders. On the other hand, the twin-screw extruder is used mainly for mixing two or more materials. As twin-screw extrusion provides uniform and homogenous mixing, the twin-screw extrusion process can be easily scaled up and optimised [2].

There are three groups of critical parameters in FDM that can condition the success of the process: machine-specific parameters, function-specific parameters, and material-specific parameters. Functioning parameters, such as the speed and the printing temperature, can be easily modified. The machine parameters depend on the brand and model of the printer (precision of the 3D printer, a disposable platform for print initialisation, or a calibration method, among others). Regarding the material, the majority of the pharmaceutical-grade polymers do not have ideal properties for being processed by FDM, mainly due to rheological and printer feeding issues [3]. The polymers used in FDM that have proven to be suitable for 3D printing, despite having good mechanical properties, present with high melting points; therefore, their usage may be inadequate for thermolabile drugs [2]. Additionally, for good feeding-speed control, it is important that the filament fits well between the two feeding rollers. If the filament is fragile, it will break within the head, and that will produce an obstruction that will not allow the filament’s progress to the heating zone. The accumulation of parts of the filament in the feed head is one of the most common problems in FDM, so the filament must be formulated with the right properties to allow it to flow through the entire feed head [3]. The production of the filaments used for the 3D printing of drugs is sometimes a slow and difficult process because they need a specific shape and diameter that could be difficult to achieve, in addition to specific mechanical and thermal properties [1].

In a 3D printing filament, one or several thermoplastic polymers will be the components in greater proportion, therefore, making them one of the key components in the mixture [6]. There are a limited number of polymer materials used as excipients in the manufacture of the filaments used in FDM. The most studied pharmaceutical grade polymeric materials for the production of these filaments are: polyvinyl alcohol (PVA), polylactic acid (PLA), polycaprolactone (PCL), ethyl cellulose (EC), hydroxypropyl methylcellulose (HPMC), polivinilpirrolidona (PVP), Soluplus^®^, Kollicoat^®^ IR, and Eudragit^®^, among others [1,7] It is possible that, despite choosing the right polymer for the desired liberation, the mechanical properties of the filament are inappropriate for the printing process. Due to this, it is desirable to mix different polymers with different release properties as well as good mechanical properties. In addition to the polymers and the drug concerned, it is common to use auxiliary excipients such as plasticizer, lubricants, antioxidants, and fillers [1,6,8]. The choice of the filament components is a crucial step when designing the pharmaceutical dosage form using FDM 3D printing.

It has been proven that the addition of plasticizers make the polymer behave better by reducing the glass transition temperature and by improving flow properties [3]. Specifically, it reduces the filament brittleness—which is really important in FDM—and also reduces the viscosity, which improves the material flow and enables lower-temperature processing for thermolabile drugs [9]. It is important to keep in mind that an excess of plasticizer can produce feeding defects by becoming too ductile. In that case, an appropriate level of plasticizer is crucial. Ideally, the filament should have some flexibility, allowing it to bend without losing its shape when the force applied to it is released [3].

Inconsistent mix flow is another problem when producing filaments. In order to ensure good flow properties, lubricating agents such as microcrystalline cellulose or magnesium stearate are used [10]. Lubricants will lower the temperature during the extrusion process because they reduce the friction forces generated by the extrusion screws [11]. Additionally, the addition of lubricants ensures a uniform exit of the filament through the nozzle because it improves the flow of the mixture by reducing the friction from the screws, giving the filaments their appropriate diameters [10]. Inconsistency in diameter can result in a change in the desired dose of the printed dosage form, an uneven thickness of deposited layers, and variations in release properties. Inconsistencies in filament diameters are often due to a phenomenon known as die expansion or extrudate expansion, in which the extruded filament expands after exiting the extruder nozzle. This is because the polymer is subject to a flow-speed change upon entering the nozzle and rearranges once it has exited [9]. Another problem could be the roughness of the filament, known as sharkskin. This is a sign of instability in the flow, probably due to a lack of homogeneity in the components’ distribution. This can be solved by increasing the temperature or adding lubricants [1].

Regarding the presence of the drug, this can be found as a solid solution or suspension-type presence in the filament after the extrusion process [12,13]. An important aspect when formulating filaments is to decide the amount of drug that should be used in order to have optimal extrusion and printing properties, since these can modify the plasticity of the filaments and the drug release profile. When the drug has a high miscibility with the polymer, the viscosity decreases—this miscibility is important for good rheological characteristics [6]. The drug dissolved in the polymer is going to act as a plasticizer, reducing the viscosity and the glass transition temperature. On the other hand, the presence of a non-molten drug affects the mechanical and rheological properties of the mixture during the extrusion process, and the drug-loaded filaments’ obtained characteristics. With a high content of non-molten drug, the filaments can be too brittle and cannot withstand the printer processing conditions (tension, bending, and compression); larger particles can also clog the nozzle and result in a viscosity that is too high [9].

For all these reasons, it is necessary to know and study the factors and properties that affect the adequate printability of the filament, carrying out a control throughout the process, from its formulation to its printing. Detecting inconsistencies in its properties before printing will allow correction of the defects produced during the process, optimizing the final pharmaceutical form as much as possible.

The objective of this work was to evaluate the extrusion process and the physical appearance of filaments made of a polymer based on hydroxypropylmethylcellulose (HPMC) Affinisol™ 15LV as a support and a non-molten model drug (metformin). For this purpose, the prepared filaments were characterised, and the influence of the auxiliary excipients was studied. Another objective of the present work is the evaluation of the printability of the obtained filaments using the FDM 3D printing process. Furthermore, a mathematical approach, including a fractal analysis, was employed to investigate the extrusion process.

## 2. Materials and Methods

### 2.1. Materials

The base polymer in the extruded filaments was hydroxypropyl methylcellulose (HPMC) Affinisol™ 15 LV (AFF), a water soluble, amorphous polymer that was kindly donated by The Dow Chemical Company (Midland, MI, USA). Metformin hydrochloride (MET) has been used as model drug and was donated by Pharmhispania S.A. (Barcelona, Spain). Magnesium stearate (MS; Fagron Iberica, Terrassa, Spain) was used as lubricant. Polyethylene glycol 6000 (PEG 6000; Acofarma, Madrid, Spain) and triethyl citrate (Sigma-Aldrich, Schnelldorf, Germany) were used as added excipients. PEGs were used as plasticizers and to facilitate the extrusion process. Triethyl citrate (TEC) was used as plasticizer too. All percentages are based on *w*/*w* unless otherwise noted.

### 2.2. Methods

#### 2.2.1. Preparation of Physical Mixtures

The physical mixtures used for the filament extrusion were made by weighing out the samples. Initially, some components had to be ground-processed before mixing. PEG 6000 was ground by milling (Retsch, type ZM 200) to obtain a smaller particle size, due to the use of components of a similar particle size that enabled the formation of a homogeneous mixture. Then, MET and PEG 6000 were separately passed through a 180 μm sieve before mixing to break down agglomerates and to ensure a homogenous particle size distribution. Finally, raw materials were manually mixed using a “geometric dilution” protocol and a mortar and a pestle until a homogenous physical mixture was obtained. Table 1 shows the different preliminary blends that were prepared. All the batch sizes were 30 g. The blends of drug and excipients were kept in a vacuum desiccator for 24 h before the extrusion process.

#### 2.2.2. Extrusion Processing

The mixtures of drug and excipients were extruded using a single-screw filament extruder (Noztek Pro Desktop Filament Extruder, Noztec, Shoreham-by-Sea, UK) in order to obtain the drug-loaded filaments. The extruder was operated at 33 rpm and the mixtures were extruded at 130–170 °C through a 1.75 mm die. The heat soak time of the extruder was set to 15 min to establish a thermal equilibrium prior to processing. The extruded filaments were stored in appropriate packaging to avoid any water uptake until printing.

#### 2.2.3. Differential Scanning Calorimetry

Differential Scanning Calorimetry (DSC) was used to investigate the thermal behaviour of the materials and formulations, as well as to confirm the compatibility of the drug and the excipients. The pure substances and filaments were studied using a DSC Q20 V24.11 Build 124. The samples of approximately 8–12 mg were placed in crimped hermetic aluminium pans. The samples were heated in an atmosphere of 100 mL/min at a ramp rate of 5 °C/min. The starting temperature was 40 °C and the end temperature was 300 °C for all the samples. Data were analysed at the Functional Characterization Service of the CITIUS in the University of Seville using a TA Instruments Universal Analysis V4.7A. The thermograms were analysed to detect thermal events.

#### 2.2.4. Mechanical Property Testing of Filaments

The mechanical properties of filaments were evaluated using a Texture Analyser TA-XT (Stable Micro Systems, Godalming, UK) at room temperature. A three-point bend (3PB) test was applied to measure the brittleness of the filaments. The filament samples were cut into 7 cm pieces, and their diameter was measured by a digital micrometer (Comecta, SA, Barcelona, Spain). The test was performed in triplicate for each filament.

Samples were placed on top of the three-point-bending holder. The trigger force was set to 4.2 g. The support span was set to 30 mm and the test speed was set to 10 mm/s. A maximum deflection from the centre of the filament, i.e., the fracture distance, was set to 10 mm. The maximum force and fracture distance were recorded by Texture Analyzer software, Texture Expert v.1.22 (Stable Micro Systems, Godalming, UK). The area under curve (AUC) and maximum stress were calculated using the Macro program in Texture Analyzer software.

#### 2.2.5. Image Processing and Fractal Dimension Analysis

The irregularity degree of the filaments’ surface can be highly abstracted into a fractal dimensional value (Df). Images of the filaments’ surface were captured with a Nikon SMZ800N (Nikon Instruments Inc., New York, USA). Each picture of 1936 × 1380 pixel resolution from a magnification factor of 1 to 8 was converted to a binary image using the Nikon software (NIS-Elements BR 5.20.02 software, Nikon Instruments Inc., New York, NY, USA) at both a 800 and 80 size scale. Black and white pictures were used, and a white color was set as the background. Matlab R2020a was employed to perform the box counting fractal analysis [14]. Once the perimeter value of each filament surface image was obtained, the inverse value of the magnification factor and perimeter values were plotted in a graphical representation. As a result, the exponent for each correlation equation was obtained, which corresponds to the Df value. A value of 1 for Df indicates that the surface of the sample is smooth. The further the Df values are from 1, the higher the filaments’ roughness is. Three images were studied for each filament, entailing a total of 30 images analysed.

#### 2.2.6. Homogeneity Studies

For evaluating the homogeneity of filaments, sections of drug-loaded filaments were cut and weighed (approx. 125 mg). Three pieces for each filament were chosen from different spots along the filaments (initial, middle, and final sections), to ensure a uniform distribution of MET along the entire filament. Samples were dissolved in 200 mL of dissolution medium (pH = 1.2) at 37 ± 0.5 °C under magnetic stirring until complete dissolution. Aliquots (5 mL) were then filtered through 0.45 mm filters (Millipore Ltd., Tullagreen, Ireland) and diluted (1:10) with the same dissolution medium. Reference curve solutions were prepared, and the obtained curve was used to determine the MET drug loading in the filament sample solutions using a UV–Vis spectrophotometer Agilent 8453 (Santa Clara, CA, USA) at 230 nm [15,16].

The Functional Characterization Service of the CITIUS in the Universidad de Sevilla used a TA Instruments Universal Analysis V4.7A.

X-ray tomography was also performed by the X-ray Laboratory Service of the CITIUS in the University of Seville using a Zeiss Xradia 610 Versa (Zeiss, Oberkochen, Germany) to confirm the drug content results. The scan was conducted at a peak voltage of 50 kV. Filaments were scanned using no filter, an optical magnification of 4X, and a pixel size of 2 µm. Image reconstruction was performed using Reconstructor Scout-and-Scan v.16.0, 11, 592 software and exported as a 16-bit tiff file for visualization.

#### 2.2.7. 3D Printing Process

The drug-loaded filaments were test-printed into cylindrical-shaped tablets. The 3D printed systems were obtained with a REGEMAT 3D V1 printer (Regemat 3D S.L., Granada, Spain) using the Fused Deposition Modeling (FDM) technique, which is an extra extrusion that filaments suffer in a 3D printer. The software REGEMAT 3D DESIGNER was used to design 3D printed systems 6 mm in height and 12.35 mm in diameter. The 3D printer allows us to set the printing parameters to improve the printability of the filaments. Thus, different combinations of printer settings values were studied. For the model drug metformin, the degradation temperature is around 222 °C [17]. Therefore, a printing temperature of around 200 °C was chosen for all filaments based on our internal data. The printing settings were as follows: extrusion temperature 180–210 °C, bed temperature 70–80 °C, layer thickness 0.35 mm, nozzle diameter 0.4–0.5 mm, feeding rate 0.56–1 mm/s, infill and perimeter speed 4–6 mm/s, and speed while travelling 15 mm/s.

Tablets were extruded on a glass slide layer by layer, according to the digitally designed object. Extruded scaffolds were formed by 17 layers with 2 sealing perimeters for each one. Firstly, the FDM nozzle extrudes the 2 bottom solid layers. In the third layer, the extruder builds parallel lines, separated by 0.8 mm from each other. In the fourth layer, the extruder repeats the process, with the difference that the lines are built in a perpendicular direction with respect to the lower ones. Thus, a quadrilateral mesh of 0.8 × 0.8 mm is created. The process continues similarly until the 15th layer. Then, 2 additional layers are built by the FDM extruder to complete the scaffold. All the steps were automatically performed, building the structure without a drying process between them.

#### 2.2.8. Physical Tests of the Printed Systems

The dimensions and the weights of the triplicate 3D-printed tablets of each filament were measured using a digital micrometer (Comecta, SA) and an analytical balance (Sartorius, type LE225D).

#### 2.2.9. Scanning Electron Microscopy

The surface of the samples was evaluated at the Microscopy Service of the CITIUS in the University of Seville using Scanning Electron Microscopy (SEM) with a FEI TENEO electronic microscope (FEI Company, Hillsboro, OR, USA) operating at 5 kV. Samples were previously coated with a 10 nm-thin Pt layer with a Leica EM SCD500 high vacuum sputter coater.

Scanning Electron Microscopy–Energy Dispersive X-ray spectroscopy (SEM–EDX) was also used, operating at 15 kV. Samples were previously coated with a 10 nm-thin C layer.

## 3. Results and Discussion

### 3.1. Formulation of Drug-Loaded Filaments

The aim was to obtain drug-loaded filaments suitable for use in a 3D printer, with MET as a model drug and AFF as the carrier polymer. Initially, a binary mixture of 50% of MET and 50% of AFF was studied. It is important to select a temperature used in the extrusion process that is as low as possible to minimize the potential negative effects on the final product in 3D printing [2,18,19]. Barrel temperatures of 130–170 °C were selected based on the manufacturer’s recommended processing temperature for AFF of approximately 130–200 °C [20]. At the lowest temperature (130 °C), the flow speed of the filaments was very low, and their surface was very rough. This could be due to the fact that the AFF was not completely molten, so that it did not form a continuum matrix surrounding the drug. The extrusion temperature was raised in 10 °C steps up to 170 °C. The higher the temperature, the higher the flow speed was. However, the filament colour was darker at 160 and 170 °C. Prasad et al., 2019 [21] tested AFF extrusion temperatures at 150, 180, and 210 °C; they observed that the polymer extrudate became darker, and that signs of degradation were visible when increasing the extrusion temperature and screw speed. These authors concluded that the darker colour was indicative of the degradation of Affinisol™ 15LV due to a combination of thermal and shear-stress effects [21]. In the current work, the darker colour at 160 and 170 °C could also be considered as a sign of degradation of AFF. Thus, 150 °C was selected as the optimal extrusion temperature.

Although the flow rate rose with the temperature increase, various excipients were added to improve the overall processability of the filaments and their physical characteristics for FDM 3D printing. This can be achieved by the addition of a lubricant and a suitable plasticizer [3,22,23,24,25,26,27] to increase the extrusion capacity of the carrier polymer [28].

Different mixtures were screened initially (Table 1); however, most of these formulations provided filaments unsuitable for printing due to the lack of appropriate flexibility and resistance. Extrusion process parameters and filament properties are summarized in Table 2. The filaments were visually assessed for colour and clarity, as well as surface texture. As can be observed in Table 2, the flow speed of blends 1, 3, and 9 was very low, suggesting that blends without MS or with a small amount of MS show hindered filament production. Thus, batches containing at least 5% MS show an enhanced flow speed, confirming that magnesium stearate is useful in facilitating the extrusion process due to its lubricant properties [10,29]. This is in agreement with the results of Goyanes et al., 2017; 2018 [30,31].

Regarding the plasticizers, the blends with TEC presented with a high flow speed, but the obtained filaments were very fragile and, moreover, the filament obtained with blend 10 was burnt. Batches containing PEG resulted in filaments with better flexibility. However, the higher the PEG 6000 amount in comparison to the MS amount, the worse the extrusion process. This is illustrated by blends 3 and 7 having the highest differences. As can be observed in Table 2, these blends could not be extruded. Finally, filaments made of blends with 5% and 7.5% *w*/*w* of both MS and PEG presented with better properties such as flow speed, flexibility, colour, and texture surface. Thus, it was found that the optimisation of the formulation with the use of plasticizer and lubricants in the correct ratios was crucial to enhance the physical characteristics of the filaments [23,31,32,33].

It has been possible to extrude these formulations with a low percentage of plasticizers and lubricants, obtaining a high load of non-molten drug. Other studies have used metformin as a model drug to obtain filaments with a high drug load [15,26]. However, they used milled metformin, and thermoplastic polyurethane was selected as the main polymer [15]. Metformin hydrochloride was also used in Gioumouxouzis et al., 2018 [26]. Nevertheless, a combined mixture of hydrophobic polymers (Eudragit RL PO and PLA) was used as the main carrier. Moreover, they had to add some plasticizer (5% PEG 400) to successfully obtain 50% metformin drug-loaded filaments. On the other hand, AFF was used as the main carrier to obtain solid dispersion drug-loaded filaments [21,34], and the addition of plasticizers was needed to obtain the filaments [34]. Kadry et al., 2018 [35] made filaments with AFF, but the non-molten drug load reached only 10% without the addition of other excipients. Therefore, the present study is the first time that suspension-type high drug-loaded filaments made with AFF have been successfully obtained when adding a low percentage of plasticizers and lubricants.

**Figure 1:**
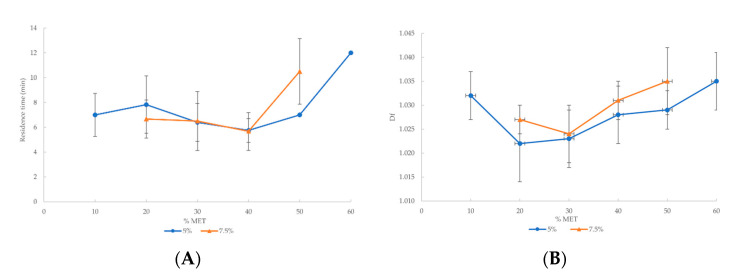

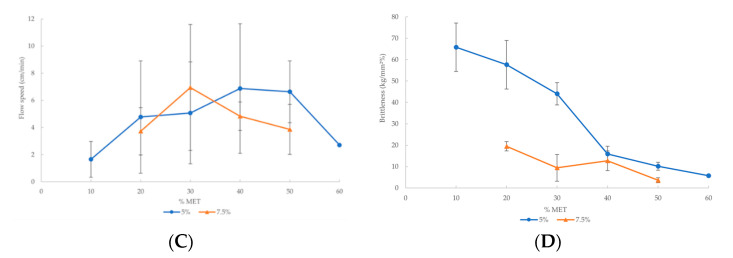
Graphical representation of the extrusion process parameters and filament parameters versus drug percentage of each filament with 5% or 7.5% auxiliary excipients: (**A**) residence time; (**B**) flow speed; (**C**) Df; (**D**) brittleness.

Once the proportions of the auxiliary excipients (MS and PEG 6000) were optimized for blends with a 50% drug load, mixtures with increasing concentrations of MET were prepared, starting from 10% and followed by 10% steps up to the maximum extrudable drug load. The results showed that drug-loaded filaments with 7.5% of both MS and PEG 6000 could be fabricated with up to 50% of MET. However, blends containing 5% of the lubricant and 5% of the plasticizer were extrudable with up to 60% of metformin. Above these drug loads the filaments could not be extruded. This might be due to the fact that the levels of AFF and the other molten excipients were not sufficient to surround the large amount of the solid, non-molten drug. Thus, the blends did not move along the heated barrel, and finally, they burnt. Blends that could be extruded are summarized in Table 3 and Table 4. Figure 1 shows the graphical representation of these parameters.

### 3.2. Blend Behaviour in the Extrusion Process

Critical points related to properties such as drug release and mechanical characteristics, among others, have been found in solid dosage forms [36,37,38,39]. To evaluate the behaviour, critical points, and thresholds of the blends in the extrusion process, some parameters summarized in Table 3 and Table 4 have been studied. Residence time and flow speed parameters were assessed, among others. The residence time of metformin formulations was determined after the barrel temperature was stable. The time at which blends were charged into the extruder feed port was denoted as t = 0. The time at which filament extrudate emerged from the die was noted as the residence time. As we can observe in Figure 1A, 60% MET and 50% MET batches—containing 5% and 7.5% of both auxiliary excipients respectively—show the longest residence time.

Regarding to the flow speed (Figure 1B), the low values shown by blends containing 10% and 60% MET should be highlighted. Flow speed increased in blends containing 10% to 30–40% MET (for batches containing 7.5% and 5% of auxiliary excipients, respectively). Nevertheless, the flow speed values decreased from 30–40% to 60% MET.

These results agree, showing a turning point in flow speed close to 30% MET (Figure 1B), so that the worse flow speed values correspond to the most extreme drug concentrations, i.e., to the filaments obtained with a drug content of 10% (lower end) and 50–60% (higher end). As Figure 1B shows, a remarkable decrease in flow speed could be observed for 10% MET. Furthermore, as Table 3 shows, the filament with 10% MET had the largest diameter. This could mean that 10% of the drug is insufficient to perform the extrusion properly, due to the low viscosity of the blend [1].

On the other hand, 60% MET and 50% MET batches containing 5% and 7.5% of both lubricant and plasticizer, respectively (Figure 1A,B), showed the longest residence time and the lowest flow speed. These results can be attributed to the high amount of solid drug contained in these blends, which hindered the extrusion process.

**Figure 2:**
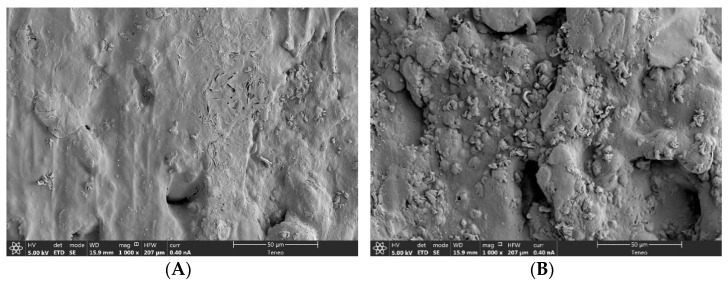
SEM images, magnification 1000×: (**A**) 40A; (**B**) 40B.

It should be also mentioned that the surface of the filaments containing 7.5% of both auxiliary excipients showed some small particles (see Figure 2). SEM-EDX was employed to investigate the nature of these particles, showing a high concentration of magnesium; this leads us to conclude that the particles correspond to magnesium stearate. Thus, it suggests MS suffered a migration to the surface [40]. This migration seems to be more evident at 7.5%. Moreover, it was observed that the higher the MET load, the higher the MS migration.

### 3.3. Physical Appearance of the Filaments

**Figure 3:**
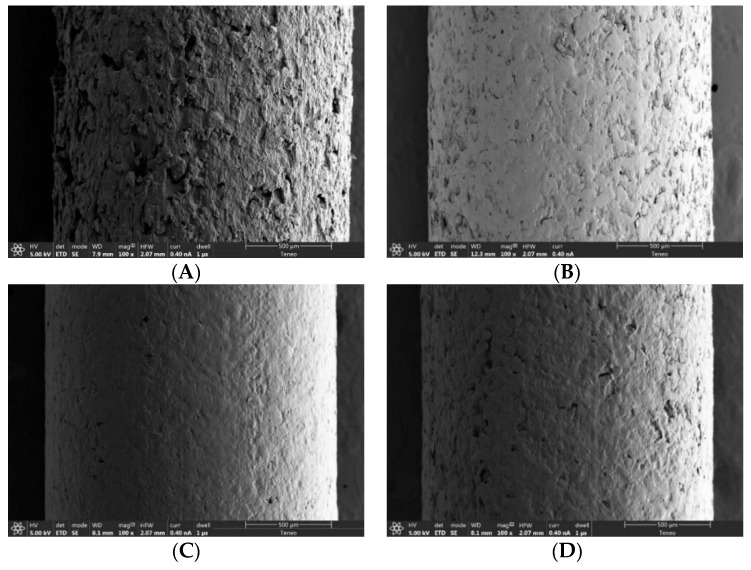

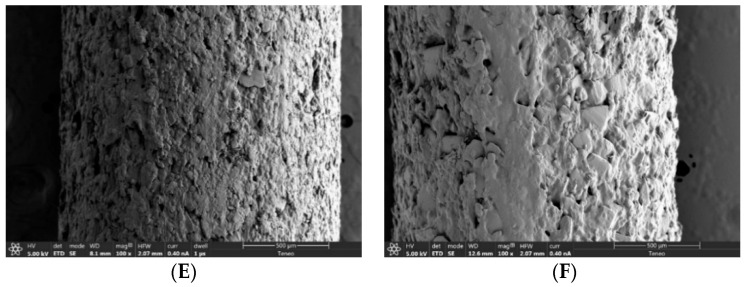
SEM images, magnification 100×, of drug-loaded filaments made with blends containing 5% of both MS and PEG 6000: (**A**) 10A; (**B**) 20A; (**C**) 30A; (**D**) 40A; (**E**) 50A; (**F**) 60A.

**Figure 4:**
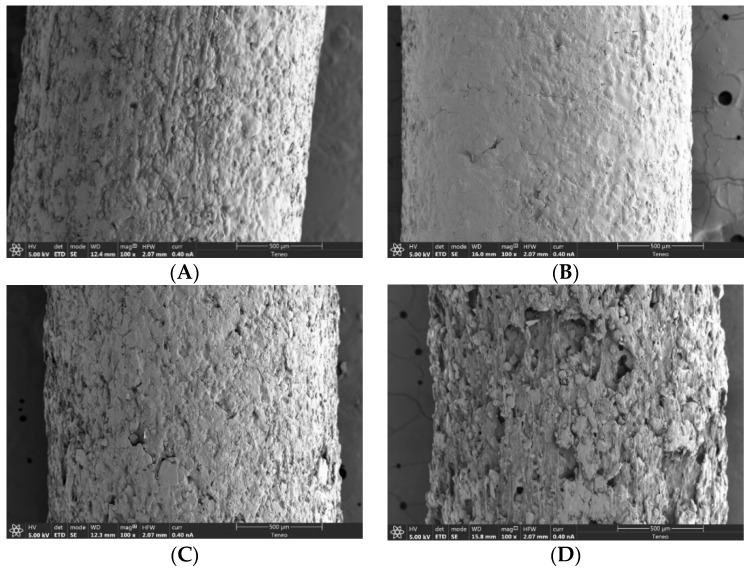
SEM images, magnification 100×, of drug-loaded filaments made with blends containing 7.5% of both MS and PEG 6000: (**A**) 20B; (**B**) 30B; (**C**) 40B; (**D**) 50B.

Roughness is a property that had not been quantified thoroughly in 3D printing filaments until Linares et al., 2021 [14]. Roughness analysis can provide valuable information regarding filament characterization. The appearance of the drug-loaded filaments is illustrated in Figure 3 and Figure 4. For the filaments made with 5% auxiliary excipients, the surface roughness apparently decreased going from 10% to 30% of the drug and increased from 30% to 60%. This suggests a mechanical threshold that agrees with the other parameters that have been discussed above. The non-molten drug seems to contribute to the stiffness and consistency of the filament.

The filament surface can be studied by applying fractal analysis. The fractal approach, based on the self-similarity concept, enables an understanding of the morphological characteristics of the filaments, and helps us to understand their behaviour in the extrusion process. Our research group is pioneering in the use of the fractal dimension [41,42,43], as well as in their application on 3D printing filaments [14]. In the present work, the fractal dimension was applied for the first time to study the roughness of hydrophilic filaments.

The fractal dimension provides a tool to objectively measure surface roughness, which can reflect the mechanical properties of the extruded filaments. According to this, the obtained fractal dimension values are shown in Table 3 and Table 4. As can be observed, there is a turning point close to 30% of MET (see Figure 1C). This critical point agrees with the previously discussed critical points corresponding to the extrusion process and filament parameters (see Figure 1).

According to this, two different behaviours can be distinguished:

(i) Above the critical point, the increase in surface roughness reflected by the fractal dimension values can be attributed to the higher friction inside the die during the extrusion process as the drug content increases [15].
(ii) Below the critical point, lower drug percentages induced flow instabilities by decreasing the viscosity, so that the flow of the material reflects small pressure changes inside the barrel due to the movement of the screw [1,44].

As Figure 3 and Figure 4 show, the SEM images fully agree with the two described trends, confirming that fractal dimensions can be used to obtain measurable objective experimental data on changes in the roughness and texture of the filaments.

A previous study of our research group found a similar critical point in polyurethane hydrophobic filaments [14]. The main difference is that in the previous study, the filaments were binary systems, i.e., they did not contain auxiliary excipients, and the roughness critical point agreed with the printability critical point. Therefore, above the critical point, the filaments could not be printed, whereas in the present study all the filaments were printed. This can be attributed to the presence of auxiliary excipients (lubricant and plasticizer) in the hydrophilic filaments of this work.

Therefore, in the filaments of the present study, the printability critical point is higher than the roughness critical point thanks to the role of the auxiliary excipients. In this respect, two findings can be emphasized: On one hand, even if the auxiliary excipients make it possible to print the filaments above the roughness critical point, this point still influences the distribution of the components in the filament, so that it can be observed by visual means.

On the other hand, in the filaments containing auxiliary excipients, the extrusion critical point is also reflected in the surface structure and the corresponding fractal dimension. As can be observed in Figure 1C, the filaments with the maximum drug loads (50% for the filaments containing 7.5% of auxiliary excipients and 60% for those containing 5%) showed the highest fractal dimensions. Furthermore, these fractal dimensions had the same value, independent of the concentration of the drug and auxiliary excipients of the polymers. The SEM images shown in Figure 3F and Figure 4D illustrate how this similar fractal dimensions corresponded to a similar roughness pattern on the filaments. Further studies are needed to determine whether this agreement between printability and fractal dimension can be extrapolated to different kinds of filaments.

### 3.4. 3-Point Bend (PB) Test Measurements on Extruded Filaments

The 3PB test was helpful for measuring the brittleness of the filaments. The results have been included in Table 3 and Table 4, expressed as (kg/mm^2^%). The higher the value shown in those tables, the lower the brittleness. As can be observed in Figure 1D, from 10% to 30%, containing 5% auxiliary excipients, brittleness is lower than filaments from 40% to 60%. Additionally, the brittleness decreased at 50% with 7.5% of both MS and PEG 6000. This suggests that the more AFF in blends, the lower the brittleness. Again, the analysis of this parameter fits in with the conclusion that there is a mechanical threshold at 30%, showing a turning point in the brittleness value.

### 3.5. Estimation of the Percolation Threshold

The results obtained showed a clear change in trend with close to 30% of the drug. All the plots in Figure 1 show a threshold between 30 and 40% of solid substance (MET in our case). This pattern could be interpreted as the existence of a percolation threshold of solid particles, corresponding to the site-percolation threshold of the simple cubic lattice (31.16% *v*/*v*) [45]. Above this percentage, the drug will be found as clusters of neighbouring solid particles extending from one side to the other sides of the matrix—the so-called percolation threshold [37,46,47]. Therefore, above this critical point, the drug increases its influence on the filament’s behaviour, resulting in important changes on all the extrusion process and physical appearance parameters assessed in this study.

### 3.6. Determination of Drug Content in Filaments

The uniformity of extruded filaments is one of the key parameters for the further development of successful 3D printed systems [48]. The concentrations of MET in the filaments are shown in Table 3 and Table 4. Drug-loaded filaments showed good agreement with the theoretical value, with standard deviations of between 0 and 4.66—thus demonstrating that homogeneous mixtures were processed.

**Figure 5:**
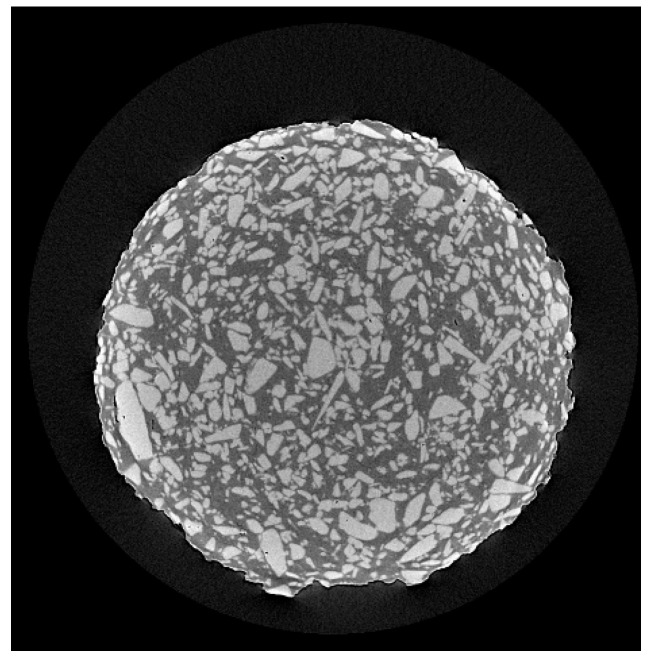
X-ray tomography image of filament with 50% MET, 40% AFF, and 5% of both auxiliary excipients.

The X-ray tomography image illustrated in Figure 5 shows the drug distribution in the filament with 50% MET, 40% AFF, and 5% of both auxiliary excipients. The lightest particles correspond to the non-molten drug (MET), whereas the darkest colour corresponds to the molten matrix of AFF, MS, and PEG 6000. As can be observed, the MET particles were homogeneously distributed in the filament matrix. Thus, the X-ray tomography image agrees with the study of the concentrations of MET and confirms that the mixing process was successfully performed.

### 3.7. Printing Filaments

**Figure 6:**
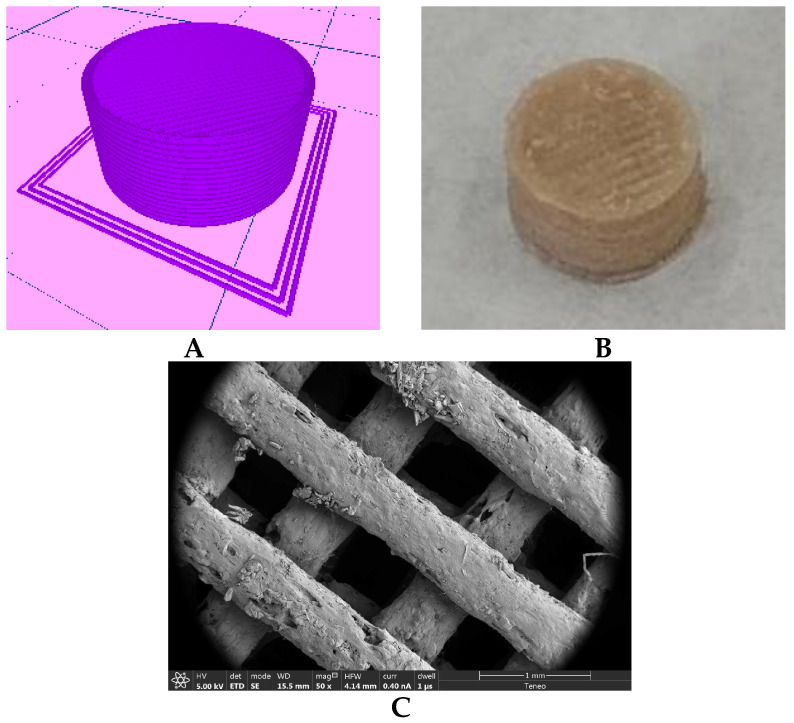
(**A**) Digital design of 3D-printed systems, showing the top solid layer and the perimeter. (**B**) 3D-printed systems made with a 30A filament. (**C**) SEM image, magnification 50×, of the internal mesh of a printed system made with a 40A filament.

Tablets were extruded according to the digitally designed object (Figure 6A).

The temperature in 3D printing has to be set higher than the temperature used in the extrusion of filaments, according to the literature [25,49].

Printing temperatures have an impact on printing quality. Appropriate printing temperatures are essential to maintain a constant flow of melts extruded through the nozzle, thereby ensuring that the melted layer is deposited onto the preceding solidified layer [25]. Different FDM temperatures were tested to estimate the optimal printing temperature for our filaments. The temperature was raised in 5 °C steps from 180 °C to 210 °C. The optimal FDM temperature for our filaments was 200 °C, based on the reproducibility and the ability of the melts extruded to stick onto the platform. This temperature was much higher than the one employed in the extrusion process (150 °C).

According to the literature, filament diameters from 1.75 ± 0.1 mm can be accepted, since they allow the drive wheels to print with accuracy [14]. All drug-loaded filaments showed a suitable diameter for printing by FDM (1.69–1.79 mm; see Table 3 and Table 4).

On the other hand, printing speed and flow speed also have an impact on printed systems’ quality [25]. Combining these parameters and varying their settings, the optimal printing speed and flow speed were 0.75 mm/s and 4 mm/s, respectively, keeping the bed temperature at 80 °C and using a 0.5 mm nozzle. These parameters made it possible to print the systems in triplicate.

These parameters were applicable for the obtained filaments, which showed very good 3D printing properties, and the printing of the systems (Figure 6B) was performed without any drawbacks. Figure 6C shows the internal mesh of the printed systems. Some drug particles can be observed on the surface of the melts extruded by FDM, although its appearance is homogeneous.

However, technical problems were found with the 60A filaments. Thus, the printing speed and flow speed had to be set at 2 mm/s and 0.5 mm/s, respectively, to improve their printability. These adjustments had an impact on the printed systems’ dimensions, increasing them. The physical characteristics of the 3D-printed systems obtained at 0.75 mm/s flow speed and 4 mm/s infill speed were: a height of 6.22 ± 0.15 mm, diameter of 13.06 ± 0.15 mm, and weight of 663.684 ± 22.36 mg. However, the physical characteristics of the 3D-printed systems obtained at 0.50 mm/s flow speed and 2 mm/s infill speed were: a height of 6.35 ± 0.22 mm, diameter of 13.4 ± 0.14 mm, and weight of 746.43 ± 18.05 mg. Yang et al., 2018 reported the influence of printing parameters on printability, demonstrating that higher printing speeds produced tablets with a better appearance, which agrees with our results.

### 3.8. Differential Scanning Calorimetry (DSC)

**Figure 7:**
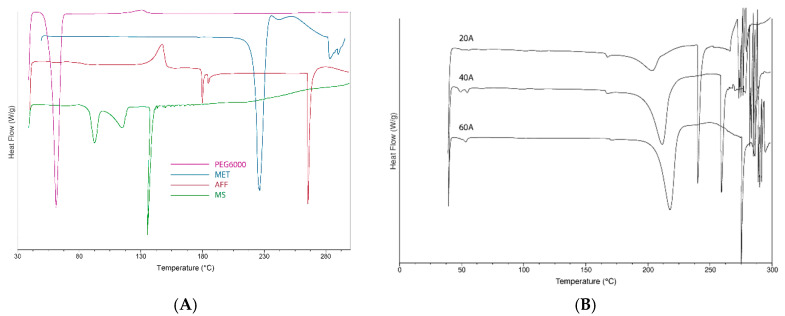
DSC thermograms of (**A**) pure materials, (**B**) drug-loaded filaments.

The thermal behaviour of pure substances and drug-loaded filaments is presented in Figure 7. The DSC thermograms in Figure 7A show the characteristic melting endotherm peak for MET (at temperature 229 °C), which is in good agreement with the literature [26]. The AFF thermogram shows an endotherm peak at 265 °C, which likely depicts the degradation of AFF [20]. However, a slight shift of the endothermic peak temperature of MET was observed with the filaments (Figure 7B) compared to that obtained with pure MET. In addition, the DSC thermogram of the filaments retained the melting endotherm peak of PEG 6000 at 60 °C [24,50] and a slight endotherm peak of MS at 130 °C (Figure 7) [51]. This suggests a potential interaction (but not necessarily an incompatibility) of MET with AFF. As seen in Figure 7B, the higher the concentration of the drug, the more prominent the melting peak for MET is.

## 4. Conclusions

High drug-loaded filaments based on a hydroxypropyl methylcellulose excipient and metformin as a model drug have been successfully obtained while adding a low percentage of other excipients (lubricant and plasticizer) using a single-screw extruder. The extruded filaments developed in this study can be successfully 3D printed into tablets. Blends containing up to 60% and 50% drug loads with 5% and 7.5% of auxiliary excipients, respectively, could be extruded. Finally, drug-loaded filaments with up to 50% drug load were suitable for use in 3D printing. Moreover, the fractal dimension was applied for the first time to study the behaviour of hydrophilic filaments.

On the other hand, all the extrusion parameters studied, as well as the fractal dimension, suggest the presence of a critical point of the solid component (the drug in our case) in the studied suspension-type filaments. This critical point, which has been found in the present paper for the first time, could be essential to understanding the behaviour of filaments containing a non-molten component—suggesting a step forward in the concept of Quality by Design, as proposed by the PAT and ICH Q8 Guidelines. Further studies should be performed to confirm this finding and to study the behaviour of filaments in which all the components are melted.

## Figures and Tables

**Table 1:** Composition of preliminary blends to be extruded in filaments. All blends contained 50% metformin.

Blend	MET (%)	AFF (%)	MS (%)	PEG 6000 (%)	TEC (%)
1	50	50			
2	50	45	5		
3	50	45		5	
4	50	40	5	5	
5	50	40	5		5
6	50	37.5	5	7.5	
7	50	35	5	10	
8	50	35	7.5	7.5	
9	50	44	3	3	
10	50	35	7.5		7.5

**Table 2:** Extrusion process parameters and filament properties of preliminary blends.

Blend	Extrusion T (°C)	Flow Speed (cm/min)	Colour	Diameter (mm)	Filament Description
1	150–160	1.1	White	1.65	Light yellowish uniform filament, rough surface
2	160	3.3	White	1.67	Light yellowish uniform filament, slightly rough surface
3	150	-	-	-	-
4	150	7.9	White	1.67	Light yellowish uniform filament, smooth surface
5	150	6.3	White	1.67	Light yellowish uniform filament, slightly rough surface
6	150	3	Beige	1.69	Brownish uniform filament, highly rough surface
7	150–160	-	-	-	-
8	150	2.6	White	1.67	Light yellowish uniform filament, smooth surface
9	150	1.8	Yellowish	1.65	Yellowish uniform filament, slightly rough surface
10	150	53.3	White	1.63	Light yellowish uniform filament, rough surface

**Table 3:** Extrusion process parameters and filament parameters of blends containing 5% of both MS and PEG 6000 with 10% to 60% MET.

Blend	MET (%)	AFF (%)	Residence Time (min)	Flow Speed (cm/min)	Brittleness (kg/mm^2^%)	Diameter (mm)	Fractal Dimension	Drug Content (%)
60A	60	30	12.00 ± 0.00	2.71 ± 0.00	5.70 ± 0.65	1.69 ± 0.00	1.035 ± 0.006	65.99 ± 4.39
50A	50	40	7.00 ± 0.00	6.63 ± 2.28	10.15 ± 1.89	1.69 ± 0.01	1.029 ± 0.004	51.22 ± 4.66
40A	40	50	5.75 ± 0.96	6.87 ± 4.77	15.93 ± 3.55	1.70 ± 0.02	1.028 ± 0.006	38.44 ± 1.74
30A	30	60	6.40 ± 1.52	5.07 ± 3.75	44.03 ± 5.22	1.71 ± 0.04	1.023 ± 0.006	30.88 ± 2.07
20A	20	70	7.83 ± 2.32	4.77 ± 4.14	57.63 ± 11.36	1.70 ± 0.04	1.022 ± 0.008	22.23 ± 0.27
10A	10	80	7.00 ± 1.73	1.65 ± 1.32	65.81 ± 11.35	1.79 ± 0.10	1.032 ± 0.005	9.76 ± 0.04

**Table 4:** Extrusion process parameters and filament parameters of blends containing 7.5% of both MS and PEG 6000 with 20% to 50% MET.

Blend	MET (%)	AFF (%)	Residence Time (min)	Flow Speed (cm/min)	Brittleness (kg/mm^2^%)	Diameter (mm)	Fractal Dimension	Drug Content (%)
50B	50	35	10.50 ± 2.65	3.86 ± 1.84	3.56 ± 1.15	1.69 ± 0.04	1.035 ± 0.007	50.80 ± 3.49
40B	40	45	5.67 ± 1.53	4.83 ± 1.05	12.71 ± 4.61	1.72 ± 0.05	1.031 ± 0.004	40.26 ± 1.66
30B	30	55	6.50 ± 2.38	6.94 ± 4.64	9.42 ± 6.24	1.68 ± 0.03	1.024 ± 0.006	30.81 ± 0.47
20B	20	65	6.67 ± 1.53	3.72 ± 1.75	10.47 ± 2.16	1.69 ± 0.01	1.027 ± 0.003	20.02 ± 1.10

## Data Availability

Data are contained within the article.
